# Immune thrombotic thrombocytopenic purpura: Spotlight on long-term outcomes and survivorship

**DOI:** 10.3389/fmed.2023.1137019

**Published:** 2023-02-28

**Authors:** Sruthi Selvakumar, Angela Liu, Shruti Chaturvedi

**Affiliations:** ^1^Dr. Kiran C. Patel College of Allopathic Medicine, Nova Southeastern University, Fort Lauderdale, FL, United States; ^2^Division of Hematology and Oncology, Mount Sinai School of Medicine, New York, NY, United States; ^3^Division of Hematology, Department of Medicine, Johns Hopkins University School of Medicine, Baltimore, MD, United States

**Keywords:** thrombotic microangiopathy, thrombotic thrombocytopenic purpura, ADAMTS13, survivorship, rare disease

## Abstract

Advances in diagnosis and treatment have dramatically improved survival of acute immune thrombotic thrombocytopenic purpura (iTTP) and iTTP has evolved from an acute fatal condition to a chronic relapsing disorder. In addition to the risk of iTTP relapse, iTTP survivors are at risk of multiple adverse health outcomes including higher than expected rates of all-cause mortality, increased rates of stroke and other cardiovascular disease, and higher rates of morbidities such as obesity, hypertension, and autoimmune disorders. iTTP survivors also report neurocognitive impairment, depression, and reduced quality of life. Women with iTTP are at risk for recurrent iTTP, preeclampsia, and other maternal and fetal complications in subsequent pregnancies. ADAMTS13 activity during clinical remission has emerged as an important targetable risk factor for iTTP relapse and other outcomes including stroke and all-cause mortality. This review summarizes current literature regarding the epidemiology and potential mechanisms for adverse long-term sequelae of iTTP, outlines current best practices in iTTP survivorship care, and highlights a research agenda to improve long-term iTTP outcomes.

## Introduction

**FIGURE 1:**
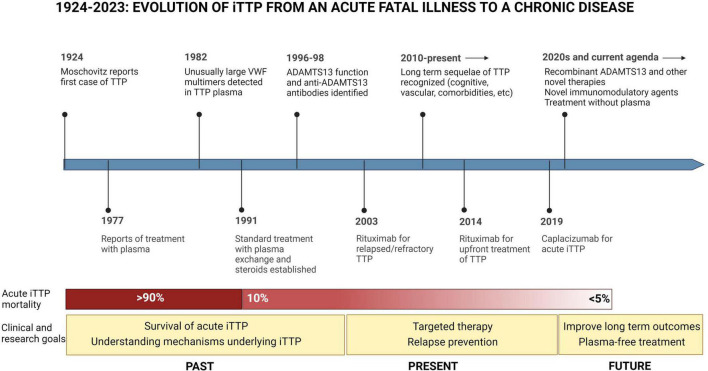
Evolution of iTTP from an acute fatal illness to a chronic disease. Since iTTP was described in 1924, the pathophysiology of iTTP has been elucidated, and advances in treatment have reduced mortality from acute iTTP episodes from over 90 to less than 5% per episode. In this landscape, the clinical and research agenda has also evolved toward a focus on issues of survivorship, and further advancements in therapy including the development of novel targeted therapies targeting immune response as well as VWF-platelet microthrombi, and steps toward treating iTTP without plasma.

**FIGURE 2:**
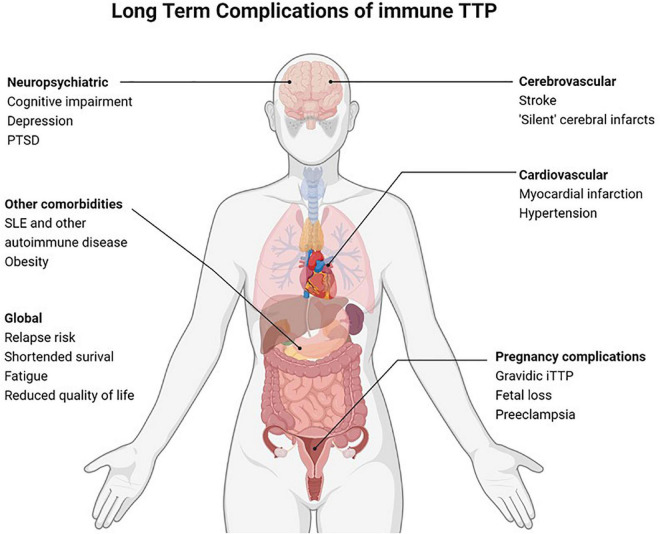
Long term complications of immune TTP.

Immune thrombotic thrombocytopenic purpura (iTTP) is a rare hematologic disorder characterized by episodes of microvascular thrombosis and ischemic organ damage (1). iTTP is caused by an autoantibody-mediated deficiency of ADAMTS13, a von Willebrand factor-cleaving protease that results in circulating high molecular weight multimers of von Willebrand factor that cause platelet aggregation and systemic microvascular thrombi (2). Untreated iTTP is almost universally fatal; however, treatment with plasma exchange (PEX) and immunosuppression has reduced mortality in acute iTTP from >90% to < 5–10% (3–5). Recent therapeutic advances, including Rituximab (6–10) and caplacizumab (11), have further improved outcomes of acute iTTP. Since most patients now survive acute iTTP, late complications and survivorship issues have emerged as an important clinical and research focus with the potential to improve outcomes for the growing number of individuals living with this rare disorder (Figure 1) (12, 13). Until recently, iTTP was viewed primarily as an acute condition, and survivors were expected to return to their previous level of health except for a 30–50% risk of relapse (14). However, recent studies suggest that iTTP survivors experience a plethora of adverse long-term health outcomes following recovery (12). These range from higher than expected rates of all-cause mortality and increased rates of chronic morbidities such as hypertension (15), obesity (12), cardiovascular disease including stroke (16–18), renal injury (19), and autoimmune disease (20) as well as neurocognitive impairment (21, 22), depression (22, 23), and reduced quality of life (Figure 2) (24). Women with iTTP may also experience recurrent iTTP as well as other maternal and fetal complications in subsequent pregnancies (25, 26).

With this paradigm shift of approaching iTTP as a chronic disorder, the long-term sequelae of iTTP, and their underlying mechanisms and risk factors are an active area of investigation. This review summarizes current literature investigating long-term outcomes after recovery from acute iTTP and current best practices in iTTP survivorship care.

## iTTP relapse

### Relapse risk

Despite successful management of most acute episodes, iTTP remains a chronic relapsing disorder. Relapse rates ranging from 30% to over 50% have been reported, and each relapse comes with the risk of significant morbidity and mortality (27–31). While most relapses occur within the first 2 years after the initial episode, relapses have been reported even after a decade (29–31). Notably, the increasing use of rituximab with the first presentation of iTTP likely delays relapse, which may then occur later in the disease course (30, 32). Multiple risk factors for relapse have been identified, including younger age, male sex, presenting in iTTP relapse (versus a *de novo* episode), non-O blood group, and Black race (30–32). However, these are generally non-modifiable risk factors.

Reduced ADAMTS13 activity during clinical remission has been identified as currently the most reliable, and importantly targetable, risk factor for relapse (33, 34). Jin et al. analyzed 157 serial measures (in 24 patients) of ADAMTS13 activity and ADAMTS13 IgG antibodies and reported that while ADAMTS13 antibody levels were not significant predictors of relapse, lower ADAMTS13 activity (*p* = 0.03) and younger age (*p* = 0.02) were significant risk factors for TTP relapse and particularly sensitive and specific for relapse in the next 90 days (34). Peyvandi et al. reported significantly lower levels of ADAMTS13 activity (12% vs. 41%, *p* = 0.007) and antigen (36 vs. 58%, *p* = 0.003) among patients with recurrent TTP compared to patients with no recurrence, and severely low ADAMTS13 levels (<10%) were associated with a higher risk of recurrence (OR: 2.9, 95% CI: 1.3–6.8, *p* = 0.01). This study additionally reported that the presence of ADAMTS13 antibodies increased the likelihood of TTP recurrence (OR: 3.1, 95% CI: 1.4–7.3, *p* = 0.006) (33). A more recent multi-institutional study also demonstrated similar findings, identifying both ADAMTS13 activity ≤ 20% and high anti-ADAMTS-13 titers as independent risk factors for iTTP relapse (35). Recognizing the significance of reduced remission ADAMTS13 as a predictor of relapse, the International Working Group in iTTP recently updated its outcome definitions to include ADAMTS13 relapse, defined as ADAMTS13 activity < 20% during clinical remission (normal platelet count and no symptoms of iTTP) (36).

### Relapse prevention

Recent studies have explored the use of rituximab to promote swifter recovery of ADAMTS13 activity, decrease ADAMTS13 antibodies, and subsequently decrease relapse rates. Early observational studies showed that patients with refractory or relapsed iTTP treated with rituximab had remarkably low rates of relapse (0–19%) (7–10). Subsequently, a phase II trial with matched historical controls showed that early use of rituximab reduced the 2-year relapse rate from 57 to 10% (28), which led to wider adoption of upfront rituximab for the treatment of acute iTTP, which is endorsed by the ISTH iTTP treatment guidelines (37). Most recently “preemptive” rituximab is often used to prevent clinical relapse in patients in clinical remission but with ADAMTS13 relapse. This is largely based on observational data from the French iTTP registry in which 92 patients with ADAMTS13 < 10% in clinical remission were preemptively treated with rituximab (38). The median cumulative relapse rate decreased from 0.33 (IQR 0.23–0.66) episodes per year before rituximab therapy to 0 (IQR 0–1.32) episodes per year after treatment (*P* < 0.001) (38). Compared with 23 historical controls with ADAMTS13 < 10% who did not receive preemptive rituximab, the relapse rate was much lower in the rituximab treated patients (15 vs. 74% *P* < 0.001) (38). Our practice is to monitor ADAMTS13 activity every 3 months during clinical remission for most patients, and offer preemptive rituximab if ADAMTS13 activity is <20–30% (13). The decision for preemptive rituximab is frequently influenced by a prior history of relapse; we recommend this most strongly for patients who have already had iTTP relapses since they are at much greater risk for relapse and most likely to benefit from preemptive rituximab. Some patients with a single episode of acute iTTP elect for observation alone. The monitoring and treatment plan can be further individualized. For example, patients with a single iTTP episode may opt for less frequent follow up at every 6–12 months starting at 2–3 years after the index episode (13); however, patients with a history of relapses or those that have needed repeated courses of preemptive rituximab will likely benefit from continued close follow up.

A large retrospective study from the United States Thrombotic Microangiopathy Registry that included 645 participants reported that Black patients had higher relapse rates, and the relapse free survival prolonging benefit of rituximab was also reduced in Black participants, particularly those with relapsing iTTP (32). Thus, Black patients may require closer monitoring, earlier retreatment, and consideration of alternative immunosuppressive therapies. Overall, about 15% of patients may be refractory to rituximab or may have only a transient response (38). In some of these cases, retreatment with an anti-CD20 directed therapy may be effective but comes with increased risk of adverse events and uncertain efficacy (39). Cyclosporine A has shown excellent efficacy in improving ADAMTS13 activity in patients refractory to rituximab (40). Plasma cell directed therapies such as bortezomib and daratumumab have also shown promise in iTTP refractory to rituximab (41–44). Other immunosuppressive therapies such as cyclophosphamide and even splenectomy have been used with success in rituximab refractory cases (45, 46). The optimal immunosuppressive regimen after rituximab failure has not been established and remains a critical unmet need in iTTP care.

## Shortened overall survival

Immune thrombotic thrombocytopenic purpura survivors are at a higher risk of premature death compared with age-, sex-, and race-matched control populations. Moreover, since most patients will survive acute iTTP, late complications, such as cardiovascular disease, are the leading cause of mortality and morbidity (12, 15, 18). In an analysis of 57 iTTP survivors from the Oklahoma iTTP registry, 19% had died over a median follow-up of 7.8 years, which was significantly higher than expected based on age- and sex-matched U.S. or Oklahoma reference populations (12, 15). Only 18% of deaths were associated with iTTP relapse and cardiovascular and cerebrovascular complications accounted for the majority (64%) of deaths (15). Subsequently, a 222 patient cohort from the Johns Hopkins University and Ohio State University also demonstrated higher all-cause mortality in iTTP survivors compared to age and sex-matched reference populations with cardiovascular disease (27.6%, 8 of 29) and iTTP relapse (27.6%, 8 of 29) being the leading causes of death (18). Several studies have identified male sex, the number of iTTP episodes, and increasing age as risk factors for early mortality among iTTP survivors (12, 18). The French iTTP registry also reported that male sex, diabetes, tobacco use, malignancy, hypertension, cerebrovascular events, dementia, and COPD were risk factors for 1-year mortality among older iTTP survivors (47). Along with the finding that cardiovascular disease is a leading cause of death, these findings suggest that it is likely that comorbidities such as obesity, hypertension, and autoimmune disease may contribute to lower survival in TTP survivors. Indeed, TTP survivors have a higher prevalence of obesity (BMI > 30), particularly morbid obesity (BMI > 40), as well as higher rates of hypertension compared to the U.S. reference population (12), and these traditional cardiovascular risk factors are well-recognized as predictors of all-cause and cardiovascular mortality (48). The combined Johns Hopkins and Ohio State data found a trend toward increased mortality with lower ADAMTS13 activity during clinical remission (18). Lending credence to this association is an observation from the Rotterdam study, a population-based study in the Netherlands, which shows that lower ADAMTS13 activity is a risk factor for cardiovascular death in the general population (49).

## Cardiovascular disease and stroke

Cardiovascular disease is a leading cause of mortality and complications among iTTP survivors (12, 17, 18). The risk of stroke during clinical remission is increased nearly fivefold compared with age and sex matched control population (13.1 vs. 22.6%) and this risk is strongly associated with incomplete ADAMTS13 recovery during clinical remission (16). A subsequent study found that major adverse cardiovascular events (stroke, non-fatal and fatal MI, and cardiac revascularization) occurred in 29% of iTTP survivors over a median follow up of 7.6 years with the first event occurring at a mean age 10–20 years younger than in the US reference population (17). Stroke was more common than myocardial infarction, similar to the pattern seen in congenital TTP cohorts, suggesting that the brain may be particularly vulnerable to iTTP-related thromboembolic events. Black race and diabetes mellitus were associated with these adverse cardiovascular events though a clear association with remission ADAMTS13 was not seen, likely due to limited statistical power (17).

In large population-based cohorts from the Netherlands, lower ADAMTS13 activity has emerged as a risk factor for coronary heart disease, stroke, and all cause and cardiovascular mortality (49–51). The hypothesized mechanism is that the lower ADAMTS13 levels leads to an accumulation of larger, more procoagulant von Willebrand Factor multimers that promote platelet activation (52), complement activation (53, 54), and accelerated atherosclerosis (50, 51, 55, 56). This may be of particular relevance among iTTP survivors who may not fully recover ADAMTS13 activity even during clinical remission (called partial ADAMTS13 remission) (36, 49). Comorbidities associated with increased cardiovascular risk such as hypertension, obesity, and autoimmune diseases are also more prevalent in iTTP survivors (12, 15). It is likely that multiple factors including traditional cardiovascular risk factors, ischemic events during acute iTTP and reduced ADAMTS13 activity all contribute to the risk of adverse cardiovascular events among iTTP survivors (16–18). Until specific strategies to mitigate cardiovascular risk in iTTP are developed, aggressive screening and management of cardiovascular risk factors is suggested, and the use of antiplatelet therapies such as aspirin may be considered especially in patients with other vascular risk factors. Current treatment strategies target ADAMTS13 activity > 20% to prevent relapse. It is possible, but as yet unproven, whether targeting higher levels will improve outcomes such as stroke and mortality, and optimal ADAMTS13 targets still need to be established.

## Other morbidities

Compared to general population controls, iTTP survivors have a higher rate of comorbidities such as hypertension (15), obesity (15), autoimmune disorders (15), and depression, which can contribute to cardiovascular disease and mortality as well as impaired quality of life. For example, in the Oklahoma iTTP registry, the prevalence of hypertension (40 vs. 23%; *P* = 0.013) and major depression (19 vs. 6%; *P* = 0.005) and was greater than expected values from the US and Oklahoma reference populations. The rate of obesity, particularly morbid obesity, was also higher in iTTP patients. Similarly high rates of hypertension, obesity, and autoimmune disease were reported from other US cohorts (4, 17), and a French study comparing 36 elderly TTP survivors (≥65 years) with 127 age-matched controls demonstrated a higher prevalence of ischemic heart disease, stroke, osteoporosis, autoimmune disorders, and hypertension among the aging TTP patients (57). Autoimmune disorders, particularly systemic lupus erythematosus (SLE), commonly coexist with iTTP. In follow up of patients from the Oklahoma iTTP registry, the period prevalence of SLE was 12% (5 of 43), which was significantly increased compared to the U.S. population estimates (0.3%, *p* < 0.001) (15). The combined Johns Hopkins and Ohio State Cohort reported a 11% prevalence of SLE with 8.8% having other autoimmune disorders, and data from French Reference Center registry showed that 21.4% (56 of 261) patients developed autoimmune conditions in association with iTTP, of which 30.4% developed the autoimmune condition(s) during an average 22-month follow-up period (20).

The factors driving the higher prevalence of these comorbidities are incompletely elucidated and likely complex. iTTP is associated with depression, which is an independent risk factor for obesity (58), and may increase cardiovascular morbidity (59). Obesity in turn is a risk factor for hypertension, cardiovascular disease, and mortality (60, 61). iTTP more commonly affects women and Black people, the same patient demographic susceptible to autoimmune conditions like SLE (62, 63). Moreover, race is not a purely biological construct and is often a surrogate for economic status, education, access to resources, and other social determinants of health that have huge impacts on the risk of developing chronic disease, as well as overall survival (64, 65).

## Cognitive impairment

Chronic cognitive difficulties, especially issues with memory and concentration, are a frequently reported concern among iTTP survivors (21, 22, 66–69). A study that evaluated neurocognitive function in 24 iTTP survivors reported that 88% (21 of 24) performed below expectations in at least 1 of 11 domains, and 17% (4 of 24) participants scored below expectations in 6–8 of the 11 domains. Compared to healthy controls, the participants’ median scores were significantly lower in 4 of 11 cognitive domains–complex attention and concentration skills, information processing speed, language generation, and memory (21). Cataland et al. reported that 63% (17 of 27) of the study participants were classified as having cognitive impairment, specifically in the visual learning and memory domains. Additionally, neurocognitive function among patients whose most recent TTP episode occurred within 1 year of the study (73%) was significantly more impaired compared to cognitive functioning in survivors whose last episode was more than 1 year prior (31%, *p* = 0.035) (68). Finally, a recent prospective study of neurocognitive function in iTTP survivors also found that over half had cognitive impairments, most commonly affecting executive function, attention and processing speed (70).

**FIGURE 3:**
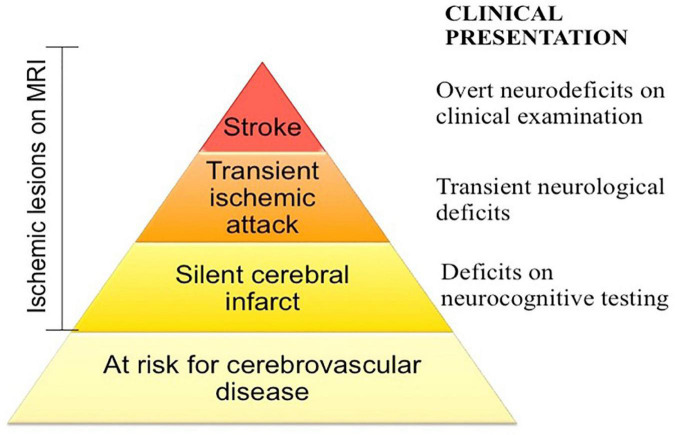
Spectrum of cerebrovascular disease in iTTP. iTTP survivors not only experience stroke at disproportionately high rates, but also exhibit evidence of silent cerebral infarction, defined as an ischemic lesion of brain magnetic resonance imaging that is not accompanied by a neurologic deficit attributable to that location of the brain, which may be associated with cognitive deficits as well. Screening for and addressing cardiovascular risk factors in at risk patients may mitigate some of the long term neurologic morbidity of iTTP.

Multiple possible etiologies for iTTP associated cognitive impairment have been proposed. Comorbid depression has been suggested as a cause based on an association of depression severity with worse cognitive performance in a European study that used self-reported cognitive performance as an outcome measure (22); however, Han et al. did not find an association of depressive symptoms among iTTP survivors in the Oklahoma registry (23). The pattern of cognitive impairments seen in iTTP is similar to that seen in patients with hypertension (71), sickle cell disease (72, 73), and other vascular disorders (74), suggesting that it could be due to acquired diffuse, subcortical microvascular lesions. In support of this hypothesis, Cataland et al. observed small and large vessel ischemic changes indicative of silent cerebral infarcts on brain MRI in 39% (9 of 23) of TTP survivors who were otherwise clinically stable and without apparently neurological symptoms, thus suggesting a higher prevalence of persistent sub-clinical neurological injury following recovery (68). A recent prospective study found that silent cerebral infarction, defined as magnetic resonance imaging (MRI) evidence of brain ischemic infarction without a corresponding neuro-deficit, was present in 50% of iTTP patients in clinical remission and was strongly associated with the presence of cognitive impairment, particularly major cognitive impairment (70). Given that silent cerebral infarctions are a risk factor for both cognitive impairment (75) and stroke (76) in the general population, it is likely that neurocognitive deficits and stroke in TTP survivors are associated with silent infarctions in the brain. Ongoing studies to establish the prevalence and incidence of silent cerebral infarcts, stroke, and cognitive impairment among TTP survivors will identify an opportunity for early interventions aimed at reducing the incidence of sub-clinical neurologic injury (Figure 3).

## Mental health and quality of life

Multiple studies have reported a very high prevalence of depressive symptoms in patients who have recovered from acute iTTP. The exact estimate varies depending upon sampling and methodology. For example, investigators from the Oklahoma registry reported a 19% point prevalence of depression in a cross sectional sample (15), but nearly 59% of patients screened positive for depression at some point during an 11 year period (23). An European observational cohort study reported that 68% (74 of 104) of iTTP survivors scored positive for depression, a significantly higher prevalence compared to the controls (*p* < 0.001), and the severity of depression did not significantly vary between a 1-year period. This study also noted a positive correlation between the survey scores for clinically relevant depression and cognitive impairment (*p* < 0.001) (22). A survey-based study conducted through the Answering TTP foundation reported that 80.8% (169 of 209) of respondents reported at least mild depressive symptoms (77). In addition, 35.1% (81 of 231) of iTTP survivors who responded to the study met the criteria for post-traumatic stress disorder (77). In this study, 21% of participants reported that they were unemployed for reasons that they attributed to iTTP sequelae (77). While this study was likely affected by responder bias that could inflate estimates, these numbers are sobering and highlight the social and economic impact of the mental health sequelae of iTTP (69).

Following recovery, iTTP survivors have described persistent difficulties with endurance, concentration, and memory, which may contribute to long-term deficits in quality of life. Lewis et al. documented that that iTTP survivors in the Oklahoma cohort scored significantly lower than the US population across all eight domains of the SF-36 questionnaire when the initial SF-36 assessment was administered 6–24 months following iTTP recovery (24). These findings were supported by Cataland et al., who reported that the aggregate mental and physical component scores of iTTP patients were significantly lower than the age- and gender-matched US norms (68).

## Pregnancy outcomes in women with a history of TTP

It is well established that pregnancy may trigger both initial and recurring episodes of congenital or immune TTP (25, 78–80). Regardless of whether the first iTTP episode is associated with pregnancy, pregnancy after a diagnosis of iTTP carries significant risk to both mother and fetus. Rates of recurrent iTTP during pregnancy range from 20% to 65%, and live birth rates range from 30% to 80% (12, 26, 80, 81). These widely varying rates are heavily influenced by the nature of the cohort (all patients followed prospectively versus high risk cases referred to tertiary care centers) and are much improved in cohorts that underwent ADAMTS13 activity monitoring and prophylactic therapy with plasma exchange when ADAMTS13 levels drop below 10–20% (81, 82). This is consistent with reports that ADAMTS13 activity at the onset of pregnancy is a predictor of relapse during pregnancy (81–84). In addition to the risk of relapse and miscarriage, upto a third of pregnancies in women with iTTP are complicated by preeclampsia, which is much higher than the 2–3% rate of preeclampsia reported in the general population (85–88). The association between iTTP and preeclampsia is likely multifactorial. Preeclampsia has been linked with reduced ADAMTS13 activity, likely due to the combination of pregnancy-related reduction in ADAMTS13, the risk of vWF-mediated placental micro-thrombosis in the setting of reduced protease activity, and the inflammation-induced inhibition of ADAMTS13 proteolytic activity (89–92). The prevalence of iTTP among Black women, who have a higher risk of hypertensive disorders of pregnancy, and the increased incidence of hypertension, which is an independent risk factor of preeclampsia, among iTTP survivors may also contribute to the increased risk of preeclampsia observed even after iTTP recovery (80, 89, 90). Though we do not have prospective studies to inform best practices for managing subsequent pregnancy in women with iTTP, these data support ADAMTS13 monitoring prior to and during pregnancy and the use of rituximab to improve ADAMTS13 activity prior to pregnancy (93).

For pregnant individuals with a history of iTTP, monitoring of platelet counts and ADAMTS13 activity is recommended at least once per trimester although our approach is to check monthly. Recurrent iTTP during pregnancy should be treated with plasma exchange and corticosteroids; rituximab may be deferred until after pregnancy given the potential effects on the fetus. If ADAMTS13 activity drops to 10–20% in the absence of thrombocytopenia and other signs of microangiopathy, low dose corticosteroids may be considered in an attempt to increase ADAMTS13 activity. If the ADAMTS13 activity drops below 10%, prophylactic plasma exchange may be considered (82). We prefer to avoid rituximab and other immunosuppressive medications in pregnancy due to concerns of safety. Low dose aspirin therapy as preeclampsia prophylaxis may also be considered (94, 95).

## Conclusion and future directions

**FIGURE 4:**
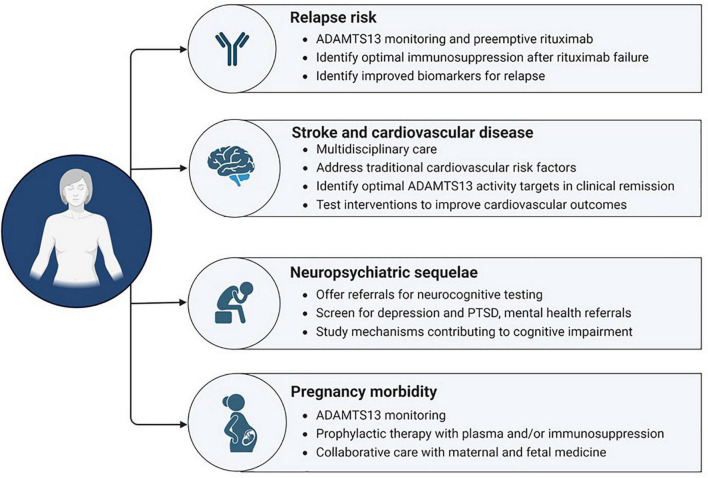
Opportunities to mitigate risk and improve long term outcomes in iTTP.

Advances in the management of TTP have dramatically improved outcomes of acute iTTP episodes, and TTP is now appropriately treated as a chronic, relapsing disorder. Other adverse health outcomes in iTTP survivors include increased mortality, high rates of cardiovascular disease, cognitive impairment, and poor mental health outcomes. In addition to traditional cardiovascular risk factors, recent data suggests that low ADAMTS13 activity is a risk factor for some of these outcomes, particularly stroke. Recent research also suggests that Black patients, who represent the majority of patients with iTTP in the United States, are at higher risk of relapse and adverse cardiovascular outcomes (17, 32). Additional research is required to understand the risk factors and mechanisms underlying these complications, to establish strategies for screening, and to identify interventions to improve outcomes that can be tested in clinical trials (Figure 4). We anticipate that the more widespread use of rituximab for first episodes of iTTP and for preemptive therapy may reduce the burden of some vascular complications to the extent that they are mediated by ADAMTS13 deficiency, although other factors are also likely to be involved. The role of recombinant ADAMTS13 in acute iTTP as well as in remission remains to be defined. As for all ultra-rare disorders, this will require multicenter studies, with national and international collaboration, and collaboration with patient advocacy groups that have facilitated rare disease research.

The future of iTTP will be focused on optimizing outcomes in survivors.

## Author contributions

SS prepared the first draft of the manuscript. AL drafted the parts of the manuscript. SC drafted the parts of the manuscript and critically reviewed and edited the entire manuscript. All authors read and approved the submitted manuscript.
